# The Human Gut Resistome up to Extreme Longevity

**DOI:** 10.1128/mSphere.00691-21

**Published:** 2021-09-08

**Authors:** Teresa Tavella, Silvia Turroni, Patrizia Brigidi, Marco Candela, Simone Rampelli

**Affiliations:** a Unit of Microbiome Science and Biotechnology, Department of Pharmacy and Biotechnology, University of Bologna, Bologna, Italy; b Department of Medical and Surgical Sciences, University of Bologna, Bologna, Italy; University of Michigan-Ann Arbor

**Keywords:** resistome, aging, antibiotic resistance, extreme longevity, metagenome, microbiome

## Abstract

Antibiotic resistance (AR) is indisputably a major health threat which has drawn much attention in recent years. In particular, the gut microbiome has been shown to act as a pool of AR genes, potentially available to be transferred to opportunistic pathogens. Herein, we investigated for the first time changes in the human gut resistome during aging, up to extreme longevity, by analyzing shotgun metagenomics data of fecal samples from a geographically defined cohort of 62 urban individuals, stratified into four age groups: young adults, elderly, centenarians, and semisupercentenarians, i.e., individuals aged up to 109 years. According to our findings, some AR genes are similarly represented in all subjects regardless of age, potentially forming part of the core resistome. Interestingly, aging was found to be associated with a higher burden of some AR genes, including especially proteobacterial genes encoding multidrug efflux pumps. Our results warn of possible health implications and pave the way for further investigations aimed at containing AR accumulation, with the ultimate goal of promoting healthy aging.

**IMPORTANCE** Antibiotic resistance is widespread among different ecosystems, and in humans it plays a key role in shaping the composition of the gut microbiota, enhancing the ecological fitness of certain bacterial populations when exposed to antibiotics. A considerable component of the definition of healthy aging and longevity is associated with the structure of the gut microbiota, and, in this regard, the presence of antibiotic-resistant bacteria is critical to many pathologies that come about with aging. However, the structure of the resistome has not yet been sufficiently elucidated. Here, we show distinct antibiotic resistance assets and specific microbial consortia characterizing the human gut resistome through aging.

## INTRODUCTION

Rates of infection with antibiotic-resistant microorganisms continue to rise worldwide (1). In Italy, 2015 data showed that over 30% of infections were caused by bacteria resistant to antimicrobial treatment for eight priority antibiotic-bacterium combinations, with more than 10,000 of 33,000 attributable deaths in Europe per year (2). Antibiotic resistance (AR) is therefore considered a critical public health threat (3). A major cause of the spread and increase in AR is the use and abuse of antibiotics (4), including those routinely used in the food industry, both for the veterinary sector and for agriculture, where they are essential in controlling the state of infestation (5, 6).

Important evidence of the impact of antibiotic misuse/abuse and environmental exposure on the development of AR was provided by the comparison of the gut resistome (i.e., the set of genes/proteins conferring AR in the gut microbiome) of Western populations with that of traditional communities (7, 8). Based on these reports, some AR genes are shared among all sampled populations regardless of lifestyle, which stresses the idea that natural environments are the first unquestionable reservoir of AR (9). However, the Western-type resistome pattern was found to support a “farm-to-fork” etiology of resistance transmission. In other words, the habitual use of antibiotics in food production and medicine in the Western world strongly affects the AR profiles of the gut microbiome, favoring the emergence of new resistances (not limited to the antibiotics to which we are exposed) and boosting their expansion through horizontal gene transfer (8, 10, 11). This has profound implications for health, because the acquisition of AR genes by the gut microbiome may also involve pathobionts, i.e., minor microbiome components with pathogenic potential, which can cause or promote disease under certain circumstances (12–14).

The relevance of antibiotic-resistant gut bacteria as an immediate and long-lasting threat to human health is well recognized, especially in compromised individuals, such as preterm infants receiving early-life antibiotics (15), elderly people with a debilitated immune system (16), and patients with cancer or autoimmune disorders (17). However, little information is available on the variation of the human intestinal resistome during healthy aging.

In an attempt to bridge this gap, here we profiled the human gut resistome of an exceptional cohort of semisupercentenarians, i.e., extremely long-lived individuals over the age of 105, compared to those of young adults, elderly, and centenarians, from a specific geographic area (Emilia Romagna region, Italy). Specifically, we characterized the type and target of resistance and related bacterial taxa in fecal samples from 62 individuals falling within the four age groups mentioned above. In addition to providing a fine characterization of antibiotic resistance within the gut microbiome of subjects at distinct times of life, our study emphasizes a progressive age-related burden in AR genes assigned to potential pathobionts.

## RESULTS

We previously identified considerable taxonomic and functional variability in the gut microbiome structure of a geographically defined cohort of 62 urban individuals, including 11 young adults (Y; mean age, 32 years), 13 younger elderly individuals (E; mean age, 73 years), 15 centenarians (C; mean age, 100 years), and 23 semisupercentenarians (S; mean age, 106 years) (18). Here, we profiled their gut resistome by analyzing 1 billion metagenomic reads from shotgun sequencing of fecal samples, with an average of 16 million (standard deviation [SD], 4 million) reads per subject. The reads were mapped to a collection of AR proteins, which summarize and organize the resistance databases of UniProt, CARD, and ARDB (see Materials and Methods). A total of 1,746 proteins were returned as best hits (from 12,567,041 mapped reads, with an average of 202,694 ± 60,145 [SD] reads per sample) and then reduced to 377 after elimination of those with fewer than 10 counts and filtering for subject prevalence of at least 40%.

### The taxonomic composition of the gut resistome along aging.

**FIG 1:**
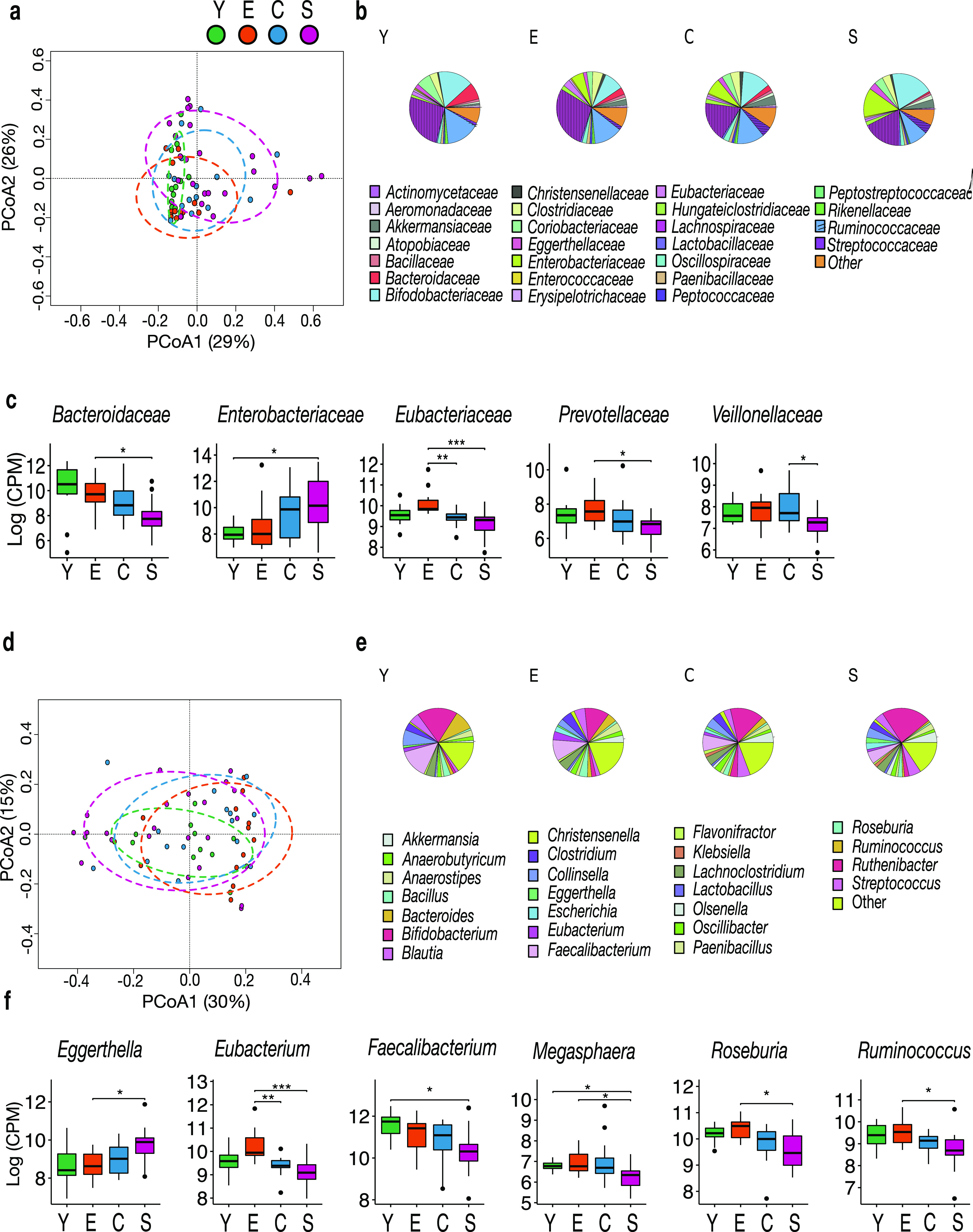
Age-related variation in the taxonomic composition of the human gut resistome. Principal-coordinate analysis of Bray-Curtis dissimilarities between the family (a) and genus-level (d) profiles of the gut resistome of young adults (Y), younger elderly individuals (E), centenarians (C), and semisupercentenarians (S). Significant separation was found at both phylogenetic levels (*P* = 0.0015 and 0.001, respectively; permutation test with pseudo-*F* ratios). Pie charts of the 25 most abundant families (b) and genera (e) for each group (Y, E, C, and S). Boxplots showing the relative abundance distribution of significantly differentially represented families (c) and genera (f) between age groups (Wilcoxon test, Bonferroni corrected *P* value). ***, *P* ≤ 0.0001; **, *P* ≤ 0.001; *, *P* ≤ 0.05.

The four age groups show separation in principal-coordinate analysis (PCoA) based on the Bray-Curtis distance between the taxonomic profiles of the gut resistome at both the family and genus levels (*P* = 0.0015 and 0.001, respectively; permutation test with pseudo-*F* ratios, vegan R package) (Fig. 1a and d). The family-level structure of the fecal resistome is dominated by a few taxa that normally abound in the human gut microbiota, i.e., *Lachnospiraceae* (mean percent counts per million [CPM] per group ± SD, 25.8% ± 9.8% in Y, 28.2% ± 13.7% in E, 17.9% ± 8.9% in C, and 17.6% ± 13.6% in S) and *Ruminococcaceae* (15.2% ± 7.2% in Y, 13.1% ± 5.6% in E, 12.6% ± 7.0% in C, and 8.5% ± 6.7% in S), as well as *Bifidobacteriaceae* (16% ± 12.4% in Y, 8.9% ± 12.8% in E, 13.0% ± 11.9% in C, and 20.5% ± 19.5% in S) (Fig. 1b). Compared to younger individuals, extremely long-lived people show decreased contribution of AR reads assigned to *Bacteroidaceae* (S versus E, *P* = 0.03, Wilcoxon test), *Eubacteriaceae* (C versus E, *P* = 0.0002; S versus E, *P* = 0.00001), *Prevotellaceae* (S versus E, *P* = 0.02), and *Veillonellaceae* (S versus C, *P* = 0.045), along with an increase in AR reads assigned to *Enterobacteriaceae* (S versus Y, *P* = 0.01) (Fig. 1c). The age-related decrease in AR reads assigned to *Bacteroidetes* members was already evident at the phylum level (S versus E and S versus Y, *P* ≤ 0.029) (Fig. S1).

10.1128/mSphere.00691-21.1 FIG S1 Relative abundance of AR reads assigned to the phylum *Bacteroidetes* during aging. Box plots showing the relative abundance distribution of AR reads assigned to *Bacteroidetes* in the gut resistomes of young adults (Y), younger elderly individuals (E), centenarians (C), and semisupercentenarians (S). An age-related decrease was found (Wilcoxon test, Bonferroni corrected; *, *P* = 0.05). Download FIG S1, EPS file, 0.06 MB. Copyright © 2021 Tavella et al. 2021 Tavella et al. https://creativecommons.org/licenses/by/4.0/ This content is distributed under the terms of the Creative Commons Attribution 4.0 International license.

At the genus level, the core resistome structure (i.e., that shared by all age groups) is essentially dominated by *Bifidobacterium* (18.4% ± 13.4% in Y, 11.0% ± 15.8% in E, 15.1% ± 13.0% in C, and 22.5% ± 21.9% in S), *Faecalibacterium* (13.9% ± 7.8% in Y, 10.0% ± 6.6% in E, 7.7% ± 4.9% in C, and 6.1% ± 7.8% in S), and *Collinsella* (7.4% ± 5.2% in Y, 3.2% ± 2.2% in E, 4.4% ± 4.6% in C, and 4.7% ± 5.6% in S), but with a decreased proportion of *Faecalibacterium*-assigned AR reads occurring with aging (S versus Y, *P* = 0.02) (Fig. 1e and f). Compared to younger subjects, the semisupercentenarian group also showed reduced contribution of AR reads assigned to *Roseburia* (S versus E, *P* = 0.03) and *Ruminococcus* (S versus E, *P* = 0.02), which are other typically health-associated, short-chain fatty acid-producing taxa (19), as well as to *Eubacterium* (S versus E, *P* = 0.00002) and *Megasphaera* (S versus E and S versus Y, *P* ≤ 0.01). On the other hand, the fecal resistome of extremely long-lived people is enriched in AR reads assigned to *Eggerthella* (S versus E, *P* = 0.01) (Fig. 1f). A linear regression analysis (with age as a continuous variable) confirmed most of the associations found, i.e., that age was positively associated with AR reads assigned to *Enterobacteriaceae* and *Eggerthella* but negatively associated with those of *Bacteroidaceae*, *Eubacteriaceae*, *Faecalibacterium*, and *Roseburia* (Bonferroni corrected *P* value ≤ 0.05) (Fig. S2). When exploring the impact of gender, we did not find a significant separation between females and males in the Bray-Curtis-based PCoA of the gut resistome at the family and genus levels (*P* ≥ 0.45, permutation test with pseudo-*F* ratios) (Fig. S3), thus suggesting a limited effect on the overall compositional structure of the gut resistome, at least in our population.

10.1128/mSphere.00691-21.2 FIG S2 Association of age with the taxonomic features of the gut resistome. Scatterplots and Poisson regression analysis of AR reads assigned at the family (a) and genus (b) level as age advances. Only the taxa that emerged in the comparison between age groups (Fig. 1) were considered. *P* values were corrected with the Bonferroni method. CPM, counts per million. Download FIG S2, EPS file, 0.4 MB. Copyright © 2021 Tavella et al. 2021 Tavella et al. https://creativecommons.org/licenses/by/4.0/ This content is distributed under the terms of the Creative Commons Attribution 4.0 International license.

10.1128/mSphere.00691-21.3 FIG S3 Impact of gender on the compositional structure of the gut resistome. Principal-coordinate analysis of Bray-Curtis dissimilarities between the family-level (a) and genus-level (b) profiles of the gut resistomes of the study subjects, stratified by gender (F, female; M, male). No significant separation was found (*P* = 0.45 and 0.47 for family- and genus-level PCoA, respectively; permutation test with pseudo-*F* ratios). Download FIG S3, EPS file, 0.1 MB. Copyright © 2021 Tavella et al. 2021 Tavella et al. https://creativecommons.org/licenses/by/4.0/ This content is distributed under the terms of the Creative Commons Attribution 4.0 International license.

### Resistome profile and age group-specific antibiotic resistance determinants.

**FIG 2:**
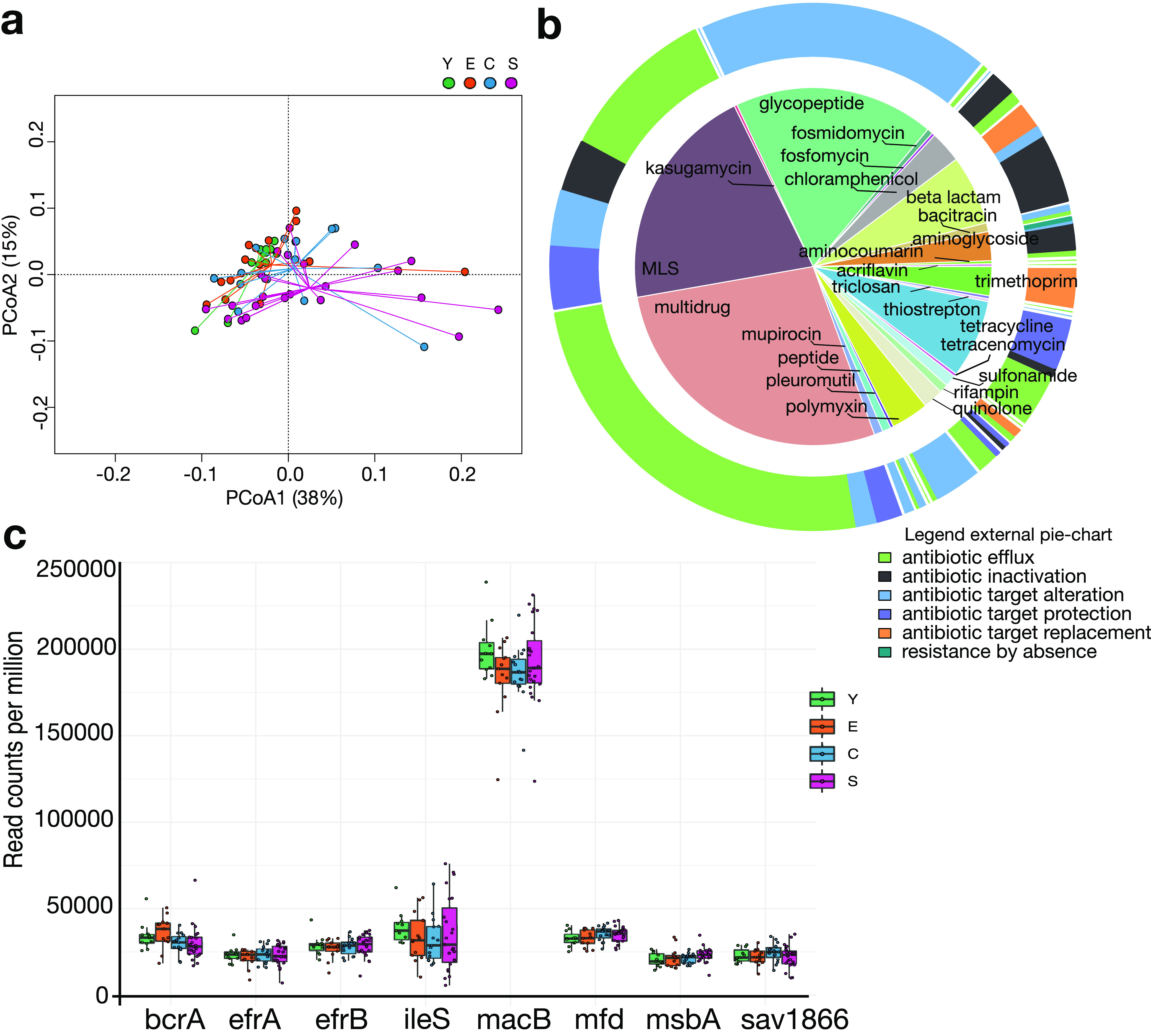
The human gut resistome across aging. (a) Principal-coordinate analysis of Bray-Curtis dissimilarities between the gut resistome profiles of young adults (Y), younger elderly individuals (E), centenarians (C), and semisupercentenarians (S). A significant separation was found (*P* = 0.012, permutation test with pseudo-*F* ratios). (b) Hierarchical pie plot, with the external pie chart depicting the resistance mechanisms of the whole gut resistome data set, while the internal donut recalls the type of antibiotics for each mechanism, with annotation obtained from the Antibiotic Resistance Ontology. (c) The core human gut resistome consists of 8 antibiotic resistance determinants with mean relative abundance above 2% across all age groups.

Consistent with taxonomic data, the Bray-Curtis PCoA on protein-level resistome profiles provides evidence of an age-related trajectory (*P* = 0.012, permutation test with pseudo-*F* ratios) (Fig. 2a), suggesting the presence of age group-specific AR determinants (ARDs). As for the resistome profiling, a summary of the results in terms of mechanisms of resistance (using Antibiotic Resistance Ontology [ARO]) and antibiotics is shown in Fig. 2b. In particular, antibiotic efflux (ARO:0010000) constitutes the prevailing resistance mechanism accounting for 44.1% of the mechanisms identified. Alteration of the antibiotic target (ARO:0001001) is the second most represented mechanism (29.1%), including both mutations and enzymatic modification of the target site. Finally, antibiotic inactivation by bacterial enzymes (ARO:0001004) is the third most represented mechanism, accounting for 12.3% of the total. A core set of 8 ARDs, with mean relative abundance above 2% across all groups, was then identified (Fig. 2c). In particular, 6 core ARDs encode ATP-binding cassette (ABC) antibiotic efflux pumps (*macB*, *bcrA*, *efrA*, *efrB*, *sav1866*, and *msbA*), 1 encodes a quinolone resistance protein (*mfd*), and 1 encodes an isoleucyl-tRNA synthetase (*ileS*) conferring resistance to mupirocin.

A total of 39 age group-specific ARDs were next identified, defined as AR determinants that were differentially represented in at least one comparison between two age groups (with log_2_ fold changes of ≤−2 and ≥2). Twenty-nine of these discriminating ARDs were annotated as coding for efflux pumps, 7 as antibiotic inactivating, 2 as antibiotic target modifying, and 1 as target protecting. The full list of age group-specific ARDs, including a description of their mechanisms, is reported in Table S2, while differential results for each age group comparison are shown in Table S3. Interestingly, compared to that of the younger group, the fecal resistomes of elderly individuals, centenarians, and semisupercentenarians show an overall larger amount of ARDs for sulfonamide (*leuO*) and multidrug, particularly *bcr*, *emrD*, *emrY*, *mdfA*, *mdtG*, *mdtL*, *robA*, and *tolC* (*P* ≤ 0.03, Wald test). Seven multidrug ARDs are specifically discriminatory for semisupercentenarians (*cpxA*, *mdtA*, *mdtB*, *mdtC*, *mdtD*, *mdtK*, and *mdtN*) (*P* ≤ 0.008).

10.1128/mSphere.00691-21.7 TABLE S2 Age group-specific antibiotic resistance determinants. For each antibiotic resistance determinant (ARD), identified by the Wald test (Bonferroni corrected *P* value ≤ 0.05), the name of the gene, the family, the resistance mechanism, and the antibiotic to which the resistance is conferred are reported. The annotation was manually curated from the Antibiotic Resistant Ontology (ARO) from the CARD database. Download Table S2, DOCX file, 0.01 MB. Copyright © 2021 Tavella et al. 2021 Tavella et al. https://creativecommons.org/licenses/by/4.0/ This content is distributed under the terms of the Creative Commons Attribution 4.0 International license.

10.1128/mSphere.00691-21.8 TABLE S3 Differential results for each age group comparison. Results from the Wald test (DESeq2) on differences in mean abundance of antibiotic resistance determinants (ARDs) between age groups. For each ARD, the mean abundance, the log_2_ fold change, the standard error, the statistically significant comparison, and the *P* value corrected with the Bonferroni method are reported. Y, young adults; E, younger elderly individuals; C, centenarians; S, semisupercentenarians. Download Table S3, DOCX file, 0.01 MB. Copyright © 2021 Tavella et al. 2021 Tavella et al. https://creativecommons.org/licenses/by/4.0/ This content is distributed under the terms of the Creative Commons Attribution 4.0 International license.

Furthermore, the semisupercentenarian group showed higher levels of ARDs conferring resistance to rifampin (*rphB*) and tetracycline (*tcr3* and *tetD*) (*P* ≤ 0.02). On the other hand, compared to that of semisupercentenarians, the resistome of young adults was enriched in ARDs for beta-lactam antibiotics (*Bl2e_cepa*, *cblA-1*, and *OXA-34*), whereas that of elderly subjects was enriched in ARDs for macrolide-lincosamide-streptogramin (*ermB* and *ermF*) (*P* ≤ 0.05).

**FIG 3:**
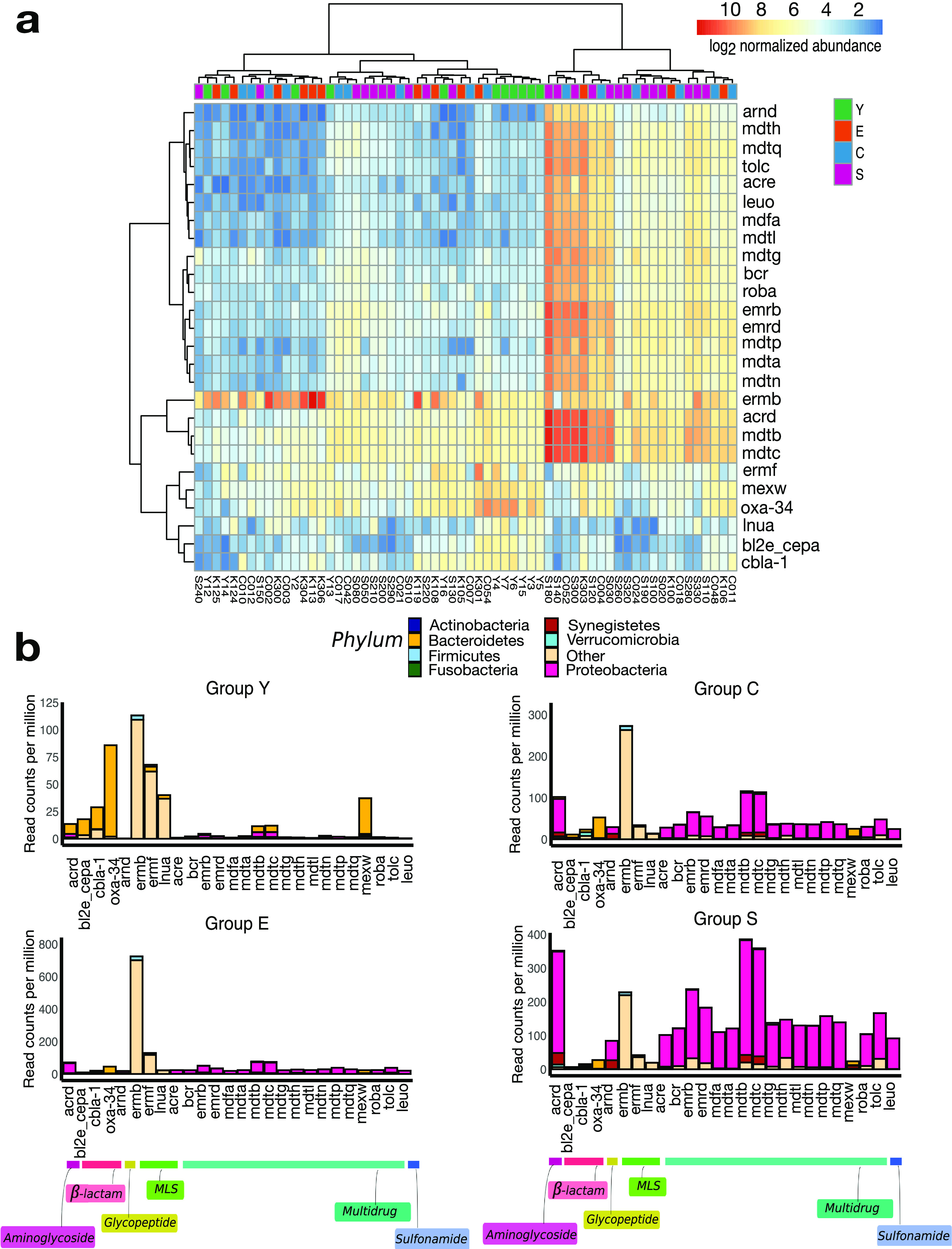
Age group-specific antibiotic resistance determinants. (a) Heat map computed on the abundances of counts normalized by library size and log_2_ scaled. The color code ranges from blue (low abundance) to red (high abundance). The samples are in columns, while the rows show the gene names of the antibiotic resistance determinants (ARDs) significantly associated with age, as identified by the three methods used (DESeq2, overdispersed Poisson generalized linear model, and Poisson regression model; Bonferroni corrected *P* value ≤ 0.05). Clustering was computed over Euclidean distances by the Ward linkage method. (b) Bar plots showing the normalized abundance (read counts per million normalized by sequencing depth) of age group-specific ARDs in the gut resistomes of young adults (group Y), younger elderly individuals (E), centenarians (C), and semisupercentenarians (S). Phylum-level assignments of ARD reads are also shown. ARDs are organized by drug to which resistance is conferred.

An overdispersed Poisson generalized linear model on the ARD count data allowed the identification of 18 significant ARDs (Table S4), in accordance with the results obtained with the Wald test (DESeq2) (Table S3). Furthermore, we tested a Poisson regression model with ARD count data and age (as a continuous variable) and identified 25 ARDs significantly associated with age (Fig. S4 and Table S5), confirming as many ARDs obtained by the Wald test (DESeq2). In total, we retained 26 ARDs (*acrE*, *arnD*, *Bl2e*_cepa, *cblA*-1, *emrD*, *ermB*, *leuO*, *lnuA*, *mdfA*, *mdtG*, *mdtH*, *mdtL*, *mdtP*, *mdtQ*, *mexW*, *OXA*-34, *robA*, *tolC*, *mdtA*, *mdtB*, *mdtC*, *mdtN*, *ermF*, *bcr*, *acrD*, and *emrB*), validated by at least two of the three methods presented (DESeq2, Poisson regression model, and overdispersed Poisson generalized linear model) (Fig. 3a). The main results discussed above were confirmed, except for ARDs conferring resistance to rifampin and tetracycline.

10.1128/mSphere.00691-21.4 FIG S4 Trend plots showing the fitted Poisson regression model between age as a continuous variable and antibiotic resistance determinants. Plots, computed with the ShotgunFunctionalizeR package, are shown for the 25 antibiotic resistance determinants (ARDs) found to be significantly associated with age (Table S4). For each ARD, age is shown in years on the *x* axis while the normalized relative frequency of the ARD is on the *y* axis. Download FIG S4, PDF file, 2.8 MB. Copyright © 2021 Tavella et al. 2021 Tavella et al. https://creativecommons.org/licenses/by/4.0/ This content is distributed under the terms of the Creative Commons Attribution 4.0 International license.

10.1128/mSphere.00691-21.9 TABLE S4 Validation of age group-specific antibiotic resistance determinants by overdispersed Poisson generalized linear model analysis. For each antibiotic resistance determinant (ARD), the comparison between age groups, the coefficient, the direction of the coefficient (and whether the DESeq2 datum is confirmed or not), the *P* value, and the antibiotic to which the resistance is conferred are reported. *P* values were corrected with the Bonferroni method. Y, young adults; E, younger elderly individuals; C, centenarians; S, semisupercentenarians. Download Table S4, DOCX file, 0.02 MB. Copyright © 2021 Tavella et al. 2021 Tavella et al. https://creativecommons.org/licenses/by/4.0/ This content is distributed under the terms of the Creative Commons Attribution 4.0 International license.

10.1128/mSphere.00691-21.10 TABLE S5 Results of regression of antibiotic resistance determinants and age as a continuous variable, using the overdispersed Poisson generalized linear model. The ShotgunFunctionalizeR R package and testGeneFamilies.regression function were used. For each antibiotic resistance determinant (ARD), the coefficient and the *P* value corrected with the Bonferroni method are reported. Download Table S5, DOCX file, 0.01 MB. Copyright © 2021 Tavella et al. 2021 Tavella et al. https://creativecommons.org/licenses/by/4.0/ This content is distributed under the terms of the Creative Commons Attribution 4.0 International license.

When looking at the taxonomic classification of these ARDs, we found that they were differently assigned even at the phylum level, depending on the age group (Fig. 3b), hinting at the establishment of age group-specific topological patterns in the gut resistome. In particular, we observed a higher contribution of ARDs from *Proteobacteria* (S versus Y, *P* = 0.002; S versus E, *P* = 0.02, Wilcoxon test) and a lower contribution of ARDs from *Bacteroidetes* (S versus Y, *P* = 0.01; S versus E, *P* = 0.02) in semisupercentenarians than in younger adults. It is worth noting that 46.3% of proteobacterial ARDs encode multidrug efflux pumps of the species Escherichia coli. On the other hand, the most represented ARDs in the fecal resistome of young adults are beta-lactamases from *Bacteroidetes* species (Y versus S, *P* = 0.03).

### Correlation network analysis between antibiotic resistance determinants and bacterial species.

**FIG 4:**
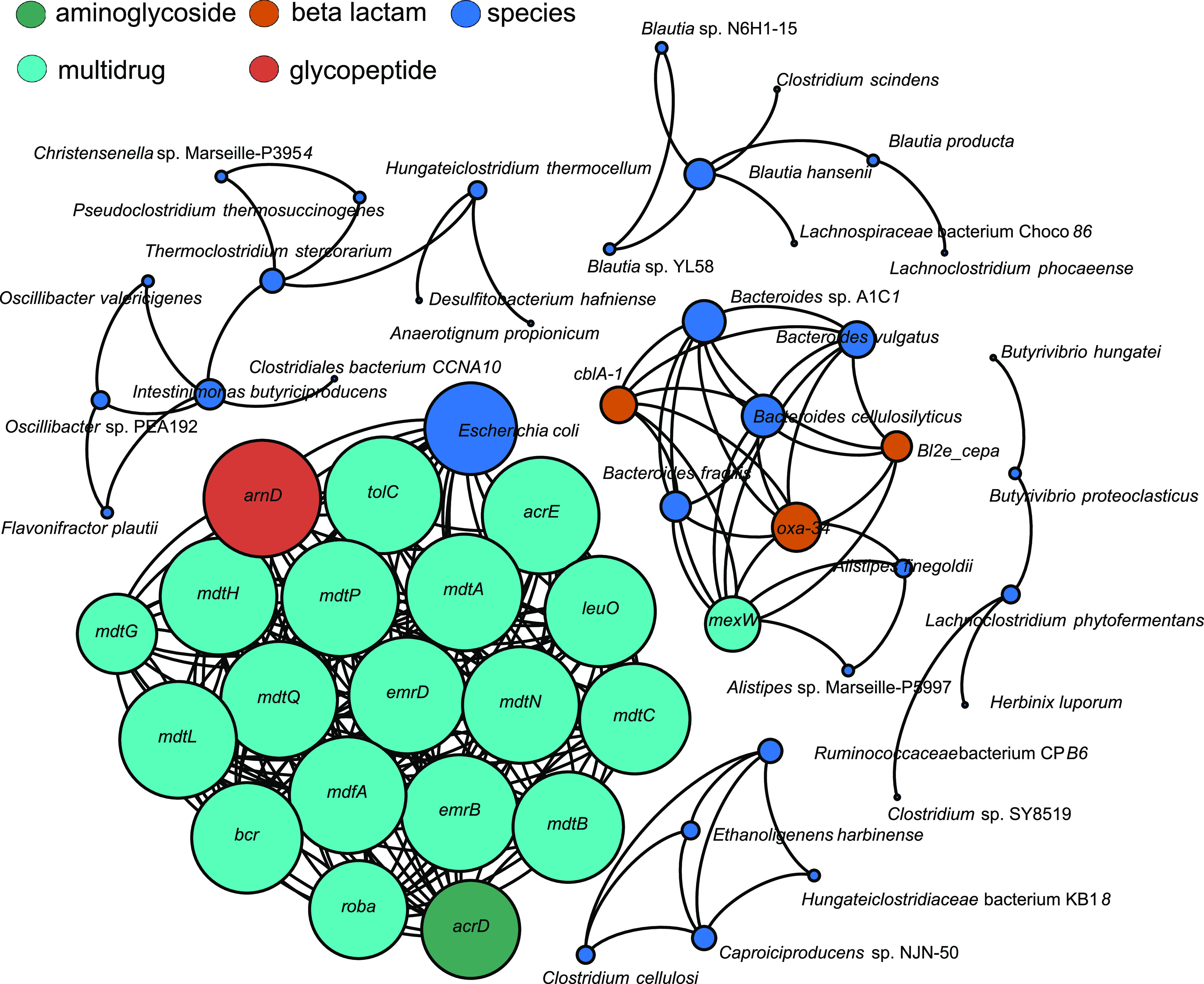
Co-occurrence network between age group-specific antibiotic resistance determinants and related bacterial species. Nodes identify ARDs (color coded by the antibiotic to which they confer resistance) or the bacterial species assigned to them. Only connected components with more than 2 nodes are depicted. The node size is proportional to the node degree (i.e., the number of edges connected to the node), and the links are Spearman correlations (see Materials and Methods).

The aforementioned findings were further highlighted by a correlation network analysis, showing associations between microbial species and ARDs. Specifically, the network in Fig. 4 shows the co-occurrence patterns between the age group-specific ADRs as identified above, and the resistome relative abundances summarized at the species level. The nodes represent the ARD entities or the species. Six connected components (i.e., nodes interconnected by edges and spatially separated by other groups of nodes and edges) with more than two nodes were identified. The highest density connected component contains 87% of nodes resistant to multidrug, of which 96% are annotated with antibiotic efflux mechanism and 4% (*arnD*) with antibiotic target protection mechanism (ARO:0001003). The AR protein families linked to E. coli are the major facilitator superfamily (MFS), accounting for 91.3% of E. coli links, and the resistance-nodulation-cell division (RND) antibiotic efflux pump family (4.3% for *acrD*). E. coli was found to have the highest diversity in terms of ARDs, with links also to determinants conferring resistance to sulfonamide and glycopeptide drugs. On the other hand, *Bacteroidetes* species, including *Alistipes* (from the family *Rikenellaceae*), are linked to beta-lactamases, with *Bl2e_cepa*, *cblA-1* and *OXA-34* accounting for 80% of the nodes, and to the RND antibiotic efflux pump family (*mexW*), accounting for the remaining 20%. The main results were validated by performing the correlation analysis with the SparCC method, using the 26 ARDs validated by at least two of the three methods in our ensemble approach (Fig. S5). Specifically, 159 significant associations from the Spearman correlation method were confirmed, with E. coli and species from the genus *Bacteroides* showing the largest number of significant connections.

10.1128/mSphere.00691-21.5 FIG S5 Correlation network results from the SparCC method. Correlations were calculated between the 25 ARDs obtained from the overdispersed Poisson generalized linear model (Table S4), of which 18 were shared with the DESeq2 method (Table S5), and the bacterial taxa at the species level. Nodes identify ARDs or the bacterial species assigned to them. Only connected components with more than 2 nodes are depicted. The node size is proportional to the node degree (i.e., the number of edges connected to the node). Nodes found with both methods (i.e., Spearman correlation using age group-specific ARDs and the SparCC method on ARDs associated with aging) are shown in yellow, and shared associations are shown with a black line between two yellow nodes. The nodes in the network obtained only from Spearman correlation are shown in grey. See also Fig. 4. Download FIG S5, EPS file, 0.1 MB. Copyright © 2021 Tavella et al. 2021 Tavella et al. https://creativecommons.org/licenses/by/4.0/ This content is distributed under the terms of the Creative Commons Attribution 4.0 International license.

## DISCUSSION

To the best of our knowledge, here we reconstructed the longest metagenomic trajectory of the human gut resistome through aging, up to extreme longevity, based on data from the gut microbiome of subjects falling into different age groups (young adults, elderly, centenarians, and semisupercentenarians). Our cohort was recruited from the Emilia Romagna region (northern Italy), and its gut microbiome was previously characterized in terms of taxonomic and functional structure (18, 20).

By cross-sectional analysis of age groups, we found that the taxonomic structure of the resistome largely overlaps with that of the microbiome, as AR-coding genes are mainly harbored by the dominant families of the gut microbial ecosystem, such as *Lachnospiraceae* and *Ruminococcaceae*, along with *Bifidobacteriaceae*. This could be the result of extensive microorganism-microorganism cross talk within the gut microbiome, with the spread of AR genes via horizontal gene transfer, potentially fueled by antibiotic exposure. On the other hand, in line with gut microbiota data (20), the resistomes of extremely long-lived people were found to be depleted in AR reads assigned to beneficial, short-chain fatty acid-producing taxa (i.e., *Faecalibacterium*, *Roseburia*, and *Ruminococcus*) (19), while they were enriched in those assigned to potential pathobionts, such as *Enterobacteriaceae* members and *Eggerthella*. These associations were also confirmed considering age as a continuous variable.

In light of the increased vulnerability of older people to infectious diseases (21), the emergence of resistant taxa could pose a serious threat to health, as well as stressing the need for resistome mapping in clinical practice, for improved efficacy of antimicrobial treatments, as has recently been discussed (17). As for resistance mechanisms, the gut resistome is mainly composed of genes conferring resistance through antibiotic efflux, along with alteration of the antibiotic target and antibiotic inactivation by bacterial enzymes. In particular, 6 ARDs involved in antibiotic efflux are similarly represented in all subjects regardless of age, likely being part of the core human gut resistome. Interestingly, through an ensemble approach utilizing analytical methods that treat age as a categorical or continuous variable, we found that aging is associated with an increasing abundance of some AR genes, including, most notably, ARDs for multidrug and sulfonamide resistance. This is especially true for semisupercentenarians, who showed the highest load of multidrug ARDs. Although aware that different subjects have been profiled at different times of life, we speculate that this may represent an adaptive response of the human holobiont to lifelong exposure to antibiotics, including those used through the food chain and for health reasons. While being a model of healthy aging, long-lived people are indeed very likely to have been more exposed to antimicrobials, also due to aging-related physiological processes, such as immunosenescence, which contributes to increased susceptibility to infections, potentially implying a greater need for medicines, including antibiotics (22, 23).

On the other hand, AR is an ancient and inherent bacterial trait that predates the human use of antibiotics (24), and AR genes are well known to be widely distributed in any environment inhabited by bacteria, including soil, air, and even household dust (25, 26). In particular, built environments have recently been appointed as an overlooked reservoir for AR, with exposure to cleaning chemicals leading to accumulation of AR genes, especially those involved in antibiotic efflux, along with loss of microbial diversity and an overall higher level of virulence (27, 28). It is thus tempting to speculate that the greater abundance of AR genes in the gut microbiomes of centenarians and semisupercentenarians, i.e., people with reduced mobility who spend more time at home (18), is the result of a top-down selection process connected not only to health status and medical history but also to lifestyle habits, including stable and constant living settings within homes, with longer and more extensive exposure to various chemicals. Consistently, we previously found that their gut microbiomes are enriched in several pathways of degradation of pervasive xenobiotics in Western societies, including those contained in common consumer and other indoor products (18). As far as the anamnesis is concerned, as expected, it was not possible to reconstruct the history of drug intake throughout the life of the subjects; however, it should be noted that only one elderly person and one centenarian were on antibiotic therapy close to sampling. This further strengthens the hypothesis that our data could be the result of a long history of antibiotic/chemical exposure and not a snapshot of what happens in the short term. However, only future studies will be able to fully define this. On the other hand, the gut resistome of young adults (who were not receiving any therapy close to sampling) was characterized by a higher abundance of AR genes for beta-lactam antibiotics, mainly harbored by *Bacteroidetes* members. As previously discussed, genes conferring beta-lactam resistance are frequently present in *Bacteroides* spp. and among the most abundant AR genes in the human gut microbiome (29, 30). Interestingly, these genes do not appear to transfer to opportunistic pathogens, such as *Enterobacteriaceae* (31), possibly explaining their poor representation in the gut resistome of older people.

In conclusion, our work, although focused only on a small Italian population from a specific geographic area (Emilia Romagna region), sheds some light on the possible trajectory of the human intestinal resistome during aging and draws attention to the potential age-related accumulation of AR genes with possibly severe repercussions on human health. Future studies in larger cohorts will ideally need to sample the same subjects over time. In any case, information on previous antibiotic exposure and medical history, over the widest possible window of time, will need to be included in an attempt to provide mechanistic explanations. Other potential confounders, such as gender, lifestyle, and geography, will need be considered to draw unambiguous conclusions about aging and resistomes. In addition to stressing the relevance of resistome surveys for more effective therapies, our results may support campaigns aimed at changing human behavior, i.e., reducing the use and abuse of antibiotics, with the ultimate goal of containing the spread of AR, thus supporting healthy aging.

## MATERIALS AND METHODS

### Subjects and study groups.

Here, we analyzed shotgun metagenomics reads of 62 fecal samples from 62 Italian subjects, generated in a previous study (18). All subjects were enrolled from the same geographic area (Emilia Romagna region, Italy). The subjects’ ages ranged from 22 to 109 years, with an average age of 85 years. In line with previous studies (18, 20), subjects were stratified into four age groups: semisupercentenarians over 105 (group S; 23 subjects), centenarians aged 99 to 105 (group C; 15 subjects), elderly individuals aged 65 to 98 (group E; 13 subjects), and younger adults aged 22 to 48 (group Y; 11 subjects). Volunteers were interviewed for the use of prescribed drugs, with particular regard to antibiotics as well as therapies for the cardiovascular system, hypertension, and metabolism (including hypoglycemic and lipid-lowering drugs). Only one elderly person and one centenarian were on antibiotic therapy in the month prior to sampling. While young adults were not receiving any therapy, some elderly individuals and most centenarians and semisupercentenarians were taking medications for the cardiovascular system and to lower blood pressure. See Table S1 for a summary of the metadata available for the study cohort.

10.1128/mSphere.00691-21.6 TABLE S1 Metadata available for the study cohort. For each subject, ID, age (and group), gender, and use of prescribed drugs in the month prior to fecal sampling, with reference to antibiotic, cardiovascular, antihypertensive, hypoglycemic and hypolipidemic therapy, are reported. Y, young adults; E, younger elderly individuals; C, centenarians; S, semisupercentenarians. Download Table S1, DOCX file, 0.02 MB. Copyright © 2021 Tavella et al. 2021 Tavella et al. https://creativecommons.org/licenses/by/4.0/ This content is distributed under the terms of the Creative Commons Attribution 4.0 International license.

### Quality assessment: read preprocessing, quality filtering, and contaminant removal.

For DNA extraction and library preparation, see reference 18. Sequencing data are available at the SRA repository (https://www.ncbi.nlm.nih.gov/bioproject/PRJNA553191).

Reads were *in silico* depleted of host DNA, using the NCBI Homo sapiens assembly 19 as a reference genome, identified with bmtagger software (ftp://ftp.ncbi.nlm.nih.gov/pub/agarwala/bmtagger) and removed with the BBMap tool (http://sourceforge.net/projects/bbmap/). Raw reads were processed with Trimmomatic (32) for adapter removal (Nextera adapters) and quality trimming. Reads were scanned by evaluating the sequences over a 4-base sliding window and setting the average quality score below Q20 for trimming. We set Trimmomatic parameters enabling reads dropping if their length was less than 35 bp. Moreover, PCR duplicates were estimated and removed with the Picard tool EstimatedLibraryComplexity (version used by the International Human Microbiome Standards project and described in their standard operating procedures). The quality of the reads was inspected before and after the preprocessing steps (FastQC) (33).

### Bioinformatics and statistical analysis.

The taxonomic classification of high-quality reads was performed with Kaiju (34) (version 1.6.3, greedy algorithm) using as a reference NCBI RefSeq (March 2019 release). On the other hand, we identified resistance proteins in our data set with Diamond (35), using the taxonomically assigned reads and the Deeparg data set (36) (14,957 entries) as a reference, which contains Swiss-Prot, Trembl (37), CARD (38), and ARDB (39). Antibiotic-resistant protein families were curated from Antibiotic Resistance Ontology (ARO) obtained with the CARD database. The member of each read pair showing at least 35% sequence identity, 80% read coverage against the hit sequence, and an E value of <0.001 was annotated as the respective mapped protein in the Deeparg data set. A table of counts was generated, summarizing the read counts per million (CPM) for each identified protein, normalized by sequencing depth. Similarly, the taxon-level CPM were computed. The taxonomic classification of reads assigned to resistant determinants was summarized at the phylum, family, genus, and species levels, retaining only taxa with a relative abundance of at least 0.1% in 20% of the data set. The Kruskal-Wallis test followed by Wilcoxon test was adopted to test for differences in relative abundance between the four age groups. Linear regression was used to evaluate the association of taxa with age as a continuous variable (lm function in R). Bonferroni correction for multiple testing was applied. A corrected *P* value of ≤0.05 was considered statistically significant.

The PCoA were obtained in R (40) with the function cmdscale (vegan package) (41), and the ordination was computed with the Bray-Curtis dissimilarities.

Age group-specific antibiotic resistance determinants (ARDs) were identified by the Wald test as implemented in the DEseq2 package (42) in R, with the design formula “∼ gender+condition,” on the AR counts mapped per sample. Hits with fewer than 10 reads across the data set were removed from the final table, as described in reference 5. Differences were assessed for each pair of groups, with proteins with log_2_ fold changes of ≤−2 and ≥2 and a *P* value threshold of 0.05 corrected with the Bonferroni correction being considered differentially abundant. The hierarchical clustering, showing the ARD relative abundances in the samples (rlog function in DEseq2), was computed with the Ward linkage method over the Euclidean distances (pheatmap package) (43). An overdispersed Poisson generalized linear model, computed with the function testGeneFamilies (ShotgunFunctionalizeR R package), was adopted to validate the results of DESeq2. A Poisson regression analysis of ARD count data and age as a continuous covariate was computed with the function testGeneFamilies.regression, from the same package.

Correlation analysis was performed between ARDs and the taxonomic profile of the gut resistome at the species level, retaining only species with a minimum relative abundance of 0.1% in 20% of the data set. The table of correlations, obtained by the Spearman method (hmisc package) (44) and retaining only associations with a value for rho greater than 0.8 and adjusted *P* values of 0.001 (Bonferroni method), was visualized as a network (R package igraph, gephi v. 0.9.2) (45). Correlations and *P* values were further computed with the SparCC method, implemented in the FastSpar software, considering 999 iterations and 999 permutations. Positive correlations with *P* values of ≤0.05 were retained (46).
