# Supplementary material for: The Human Gut Resistome up to Extreme Longevity
Source: mSphere. 2021 Sep 8;6(5):e00691-21. doi: 10.1128/mSphere.00691-21 (PMC8550338; doi:10.1128/mSphere.00691-21)
Supplement: TABLE S2 [file msphere.00691-21-st002.docx]

| **Gene** | **AR family name** | **AR mechanism** | **Antibiotic** |
| --- | --- | --- | --- |
| aad(6) | Aminoglycoside nucleotidyltransferase | antibiotic inactivation | aminoglycoside |
| acrD | resistance-nodulation-cell division (RND) antibiotic efflux pump | antibiotic efflux | aminoglycoside |
| acrE | resistance-nodulation-cell division (RND) antibiotic efflux pump | antibiotic efflux | multidrug |
| ANT(9)-Ia | Aminoglycoside nucleotidyltransferase ANT(9) | antibiotic inactivation | aminoglycoside |
| arnD | hydrolase (lipopolysaccharide biosynthesis) | antibiotic target protection | glycopeptide |
| bcr | transmembrane transporter activity | antibiotic efflux | multidrug |
| Bl2e_cepa | Beta-lactamase | antibiotic inactivation | beta_lactam |
| cblA-1 | Beta-lactamase | antibiotic target alteration | beta_lactam |
| cpxA | membrane-localized sensor kinase promoting efflux complex expression | antibiotic efflux | multidrug |
| emrB | translocase of antibiotic efflux pump: in the emrB -TolC efflux protein | antibiotic efflux | multidrug |
| emrD | antibiotic efflux pump: major facilitator superfamily (MFS) | antibiotic efflux | multidrug |
| emrY | antibiotic efflux pump: major facilitator superfamily (MFS) | antibiotic efflux | multidrug |
| ermB | antibiotic efflux pump: major facilitator superfamily (MFS) | antibiotic efflux | macrolide-lincosamide-streptogramin |
| ermF | erm 23S ribosomal RNA methyltransferase | antibiotic target alteration | macrolide-lincosamide-streptogramin |
| gadW | AraC-family regulator of mdtEF/resistance-nodulation-cell division (RND) antibiotic efflux pump | antibiotic efflux | multidrug |
| gadX | AraC-family regulator of mdtEF/resistance-nodulation-cell division (RND) antibiotic efflux pump | antibiotic efflux | multidrug |
| leuO | transcriptional activator: LySR family transcriptot factor, activator of the MdtNOP efflux pump | antibiotic efflux | sulfonamide |
| lnuA | lincosamide nucleotidyltransferase (LNU) | antibiotic inactivation | macrolide-lincosamide-streptogramin |
| mdfA | transmembrane transport | antibiotic efflux | multidrug |
| mdtA | resistance-nodulation-cell division (RND) antibiotic efflux pump | antibiotic efflux | multidrug |
| mdtB | resistance-nodulation-cell division (RND) antibiotic efflux pump | antibiotic efflux | multidrug |
| mdtC | resistance-nodulation-cell division (RND) antibiotic efflux pump | antibiotic efflux | multidrug |
| mdtD | transmembrane transporter activity | antibiotic efflux | multidrug |
| mdtG | antibiotic efflux pump: major facilitator superfamily (MFS) | antibiotic efflux | multidrug |
| mdtH | antibiotic efflux pump: major facilitator superfamily (MFS) | antibiotic efflux | multidrug |
| mdtK | transmembrane transporter activity | antibiotic efflux | multidrug |
| mdtL | transmembrane transporter activity | antibiotic efflux | multidrug |
| mdtN | antibiotic efflux pump: major facilitator superfamily (MFS) | antibiotic efflux | multidrug |
| mdtO | antibiotic efflux pump: major facilitator superfamily (MFS) | antibiotic efflux | multidrug |
| mdtP | antibiotic efflux pump: major facilitator superfamily (MFS) | antibiotic efflux | multidrug |
| mdtQ | transmembrane transporter activity | antibiotic efflux | multidrug |
| mexW | resistance-nodulation-cell division (RND) antibiotic efflux pump | antibiotic efflux | multidrug |
| OXA-34 | Beta-lactamase | antibiotic inactivation | beta_lactam |
| robA | positive regulator of acrAB efflux | antibiotic efflux | multidrug |
| rphB | phosphotransferase | antibiotic inactivation | rifampin |
| SAT-4 | Streptothricine-acetyl-transferase | antibiotic inactivation | aminoglycoside |
| tcr3 | tetracycline efflux pump | antibiotic efflux | tetracycline |
| tetD | antibiotic efflux pump: major facilitator superfamily (MFS) | antibiotic efflux | tetracycline |
| tolC | transmembrane transporter activity | antibiotic efflux | multidrug |
