# Supplementary material for: The Human Gut Resistome up to Extreme Longevity
Source: mSphere. 2021 Sep 8;6(5):e00691-21. doi: 10.1128/mSphere.00691-21 (PMC8550338; doi:10.1128/mSphere.00691-21)
Supplement: TABLE S3 [file msphere.00691-21-st003.docx]

| **ARD** | **Mean abundance** | **log2 fold change** | | **Standard error** | | **Comparison** | | **P value** | |
| --- | --- | --- | --- | --- | --- | --- | --- | --- | --- |
| aad(6) | 71.41 | -3.92 | 0.76 | | Y vs E | | 0.0001 | |  |
| acrD | 411.46 | -3.02 | 0.78 | | Y vs S | | 0.04495 | |  |
| acrE | 124.42 | -4.73 | 1.02 | | Y vs S | | 0.00158 | |  |
| ant(9)-ia | 28.19 | 3.16 | 0.80 | | E vs S | | 0.034 | |  |
| SAT-4 | 46.07 | -3.02 | 0.78 | | Y vs C | | 0.047 | |  |
| SAT-4 | 46.07 | -3.43 | 0.79 | | Y vs E | | 0.0043 | |  |
| Bl2e_cepa | 33.06 | 3.17 | 0.74 | | Y vs S | | 0.00805 | |  |
| cblA-1 | 49.27 | 2.80 | 0.73 | | Y vs S | | 0.04705 | |  |
| OXA-34 | 176.26 | 3.30 | 0.71 | | Y vs S | | 0.00165 | |  |
| arnD | 76.21 | -5.57 | 1.18 | | Y vs C | | 0.0009 | |  |
| arnD | 76.21 | -5.80 | 1.16 | | Y vs E | | 0.0001 | |  |
| arnD | 76.21 | -6.48 | 1.07 | | Y vs S | | 5.99e-07 | |  |
| ermB | 587.75 | 2.51 | 0.56 | | E vs S | | 0.003 | |  |
| ermF | 108.95 | 2.43 | 0.61 | | E vs S | | 0.028 | |  |
| lnuA | 30.51 | 2.6 | 0.69 | | Y vs S | | 0.04738 | |  |
| acrE | 124.42 | -4.82 | 1.13 | | Y vs E | | 0.0061 | |  |
| bcr | 149.78 | -3.45 | 0.85 | | Y vs C | | 0.0188 | |  |
| bcr | 149.78 | -3.34 | 0.84 | | Y vs E | | 0.0232 | |  |
| bcr | 149.78 | -4.32 | 0.76 | | Y vs S | | 6.22e-06 | |  |
| cpxA | 365.64 | -2.13 | 0.50 | | Y vs S | | 0.00805 | |  |
| emrB | 276.48 | -4.16 | 0.83 | | Y vs S | | 0.00022 | |  |
| emrD | 219.53 | -3.83 | 0.91 | | Y vs C | | 0.010 | |  |
| emrD | 219.53 | -3.75 | 0.90 | | Y vs E | | 0.0108 | |  |
| emrD | 219.53 | -4.64 | 0.82 | | Y vs S | | 5.88e-06 | |  |
| emrY | 131.45 | -4.33 | 1.06 | | Y vs C | | 0.0176 | |  |
| emrY | 131.45 | -5.45 | 1.05 | | Y vs E | | 6.62e-05 | |  |
| emrY | 131.45 | -4.98 | 0.95 | | Y vs S | | 7.74e-05 | |  |
| gadW | 88.35 | -4.37 | 0.97 | | Y vs E | | 0.0023 | |  |
| gadW | 88.35 | -3.82 | 0.89 | | Y vs S | | 0.00663 | |  |
| gadX | 128.19 | -4.69 | 1.04 | | Y vs E | | 0.0022 | |  |
| gadX | 128.19 | -4.38 | 0.95 | | Y vs S | | 0.00157 | |  |
| mdfA | 134.39 | -4.23 | 0.99 | | Y vs C | | 0.0085 | |  |
| mdfA | 134.39 | -4.35 | 0.98 | | Y vs E | | 0.0032 | |  |
| mdfA | 134.39 | -5.27 | 0.90 | | Y vs S | | 1.75e-06 | |  |
| mdtA | 152.04 | -4.07 | 0.82 | | Y vs S | | 0.00030 | |  |
| mdtB | 462.93 | -3.47 | 0.71 | | Y vs S | | 0.00044 | |  |
| mdtC | 436.49 | -3.26 | 0.73 | | Y vs S | | 0.00315 | |  |
| mdtD | 381.36 | -3.30 | 0.67 | | Y vs S | | 0.00039 | |  |
| mdtG | 189.04 | -4.13 | 0.90 | | Y vs C | | 0.0016 | |  |
| mdtG | 189.04 | -3.88 | 0.89 | | Y vs E | | 0.0041 | |  |
| mdtG | 189.04 | -5.12 | 0.81 | | Y vs S | | 9.22e-08 | |  |
| mdtH | 150.19 | -4.43 | 1.17 | | Y vs E | | 0.0490 | |  |
| mdtH | 150.19 | -5.42 | 1.06 | | Y vs S | | 0.00013 | |  |
| mdtK | 331.88 | -2.79 | 0.56 | | Y vs S | | 0.00030 | |  |
| mdtL | 156.07 | -4.81 | 1.14 | | Y vs C | | 0.0096 | |  |
| mdtL | 156.07 | -4.92 | 1.13 | | Y vs E | | 0.0042 | |  |
| mdtL | 156.07 | -5.72 | 1.02 | | Y vs S | | 1.00e-05 | |  |
| mdtN | 161.52 | -4.00 | 0.86 | | Y vs S | | 0.00148 | |  |
| mdtO | 218.85 | -4.81 | 1.08 | | Y vs E | | 0.0026 | |  |
| mdtO | 218.85 | -4.71 | 0.98 | | Y vs S | | 0.00059 | |  |
| mdtP | 199.88 | -4.24 | 1.10 | | Y vs E | | 0.0356 | |  |
| mdtP | 199.88 | -4.49 | 0.99 | | Y vs S | | 0.00254 | |  |
| mdtQ | 175.45 | -4.23 | 1.09 | | Y vs E | | 0.0323 | |  |
| mdtQ | 175.45 | -4.89 | 0.98 | | Y vs S | | 0.00028 | |  |
| mexW | 87.51 | 2.41 | 0.51 | | Y vs S | | 0.00085 | |  |
| robA | 130.43 | -3.62 | 0.85 | | Y vs C | | 0.0074 | |  |
| robA | 130.43 | -4.04 | 0.84 | | Y vs E | | 0.0004 | |  |
| robA | 130.43 | -4.51 | 0.76 | | Y vs S | | 1.37e-06 | |  |
| tolC | 186.83 | -4.46 | 1.14 | | Y vs C | | 0.035 | |  |
| tolC | 186.83 | -4.86 | 1.13 | | Y vs E | | 0.0052 | |  |
| tolC | 186.83 | -5.32 | 1.02 | | Y vs S | | 8.44e-05 | |  |
| rphB | 49.50 | -2.89 | 0.58 | | Y vs S | | 0.00025 | |  |
| leuO | 116.80 | -4.25 | 1.07 | | Y vs C | | 0.0284 | |  |
| leuO | 116.80 | -4.74 | 1.06 | | Y vs E | | 0.0023 | |  |
| leuO | 116.80 | -5.13 | 0.96 | | Y vs S | | 4.14e-05 | |  |
| tcr3 | 25.35 | -2.13 | 0.52 | | Y vs S | | 0.01906 | |  |
| tetD | 25.11 | -2.23 | 0.54 | | Y vs C | | 0.0170 | |  |
| tetD | 25.11 | -2.41 | 0.49 | | Y vs S | | 0.00036 | |  |
