# Supplementary figures and images for: The Human Gut Resistome up to Extreme Longevity
Source: mSphere. 2021 Sep 8;6(5):e00691-21. doi: 10.1128/mSphere.00691-21 (PMC8550338; doi:10.1128/mSphere.00691-21)

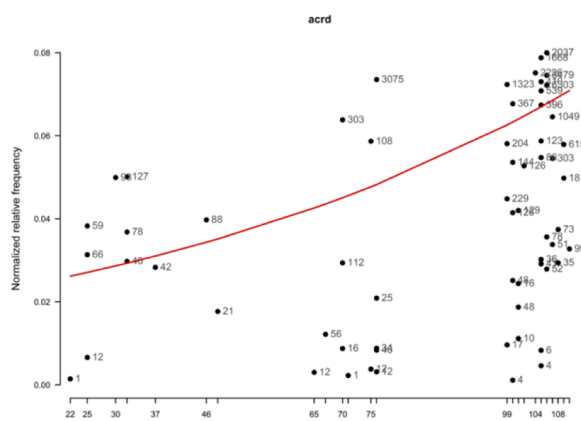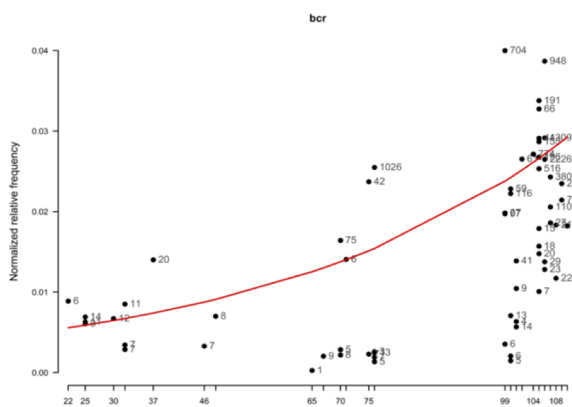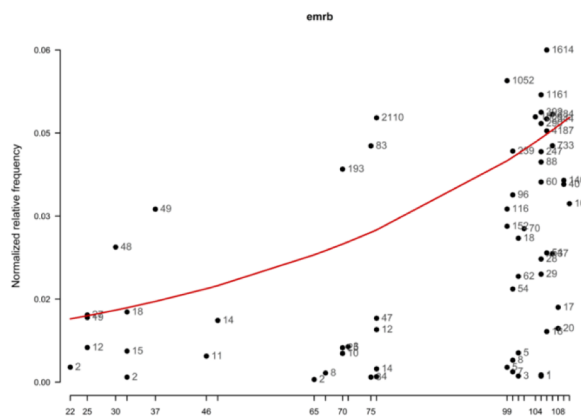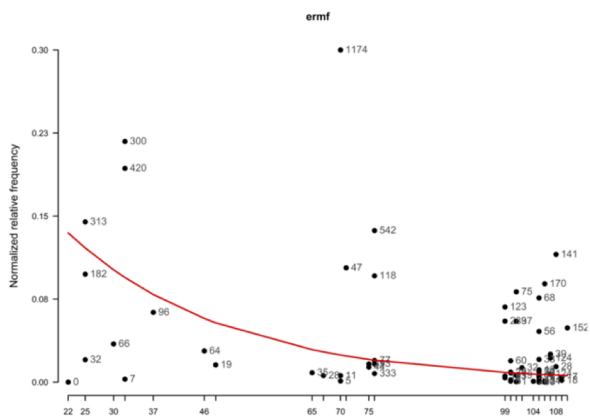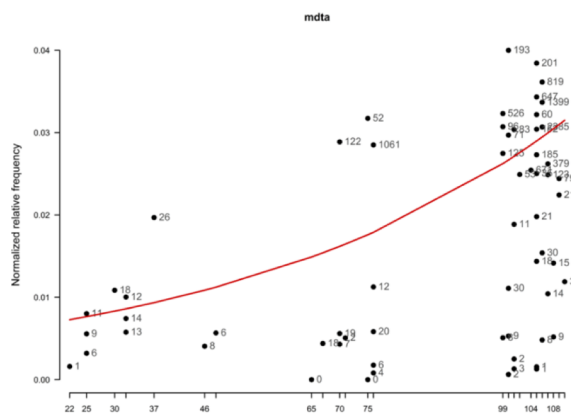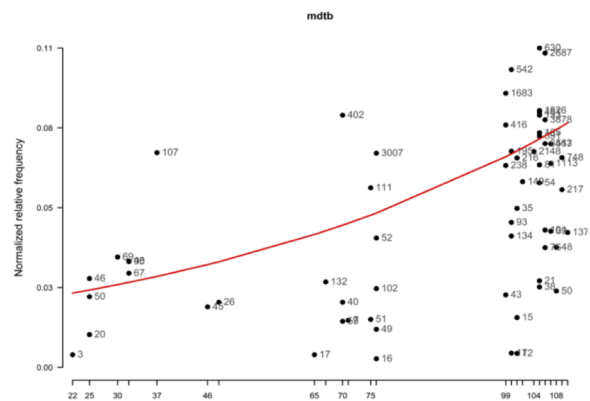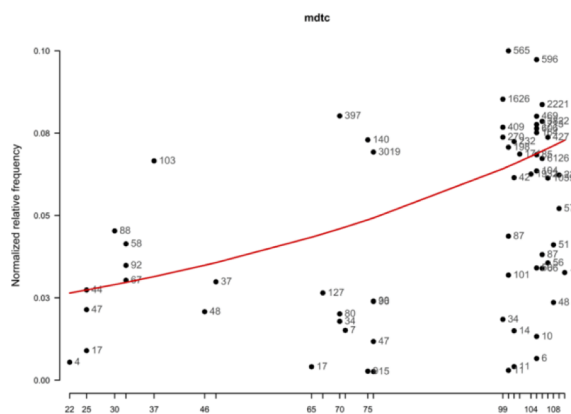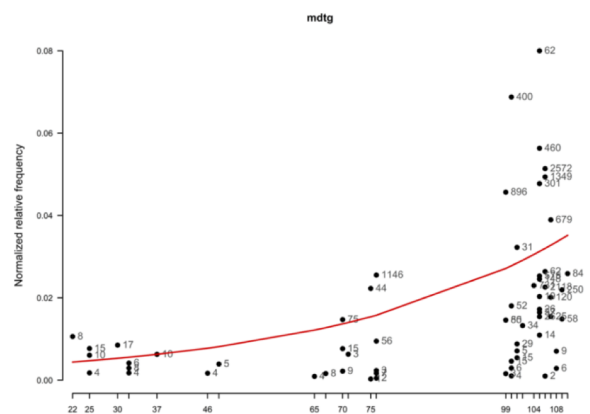

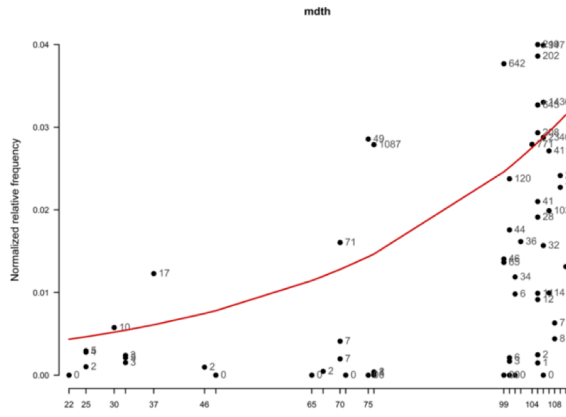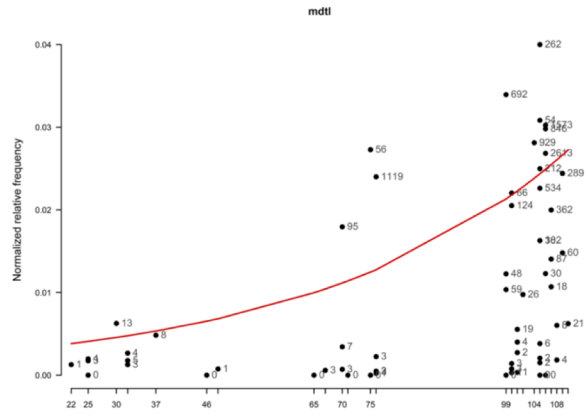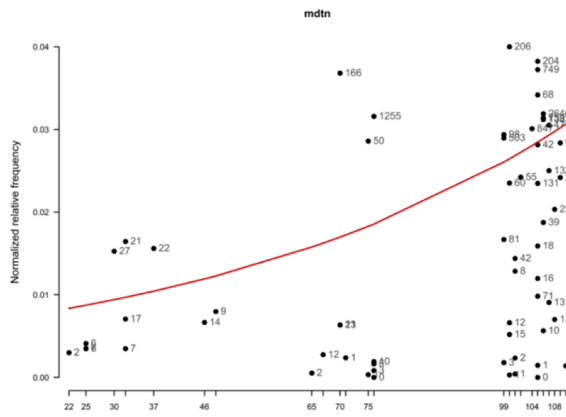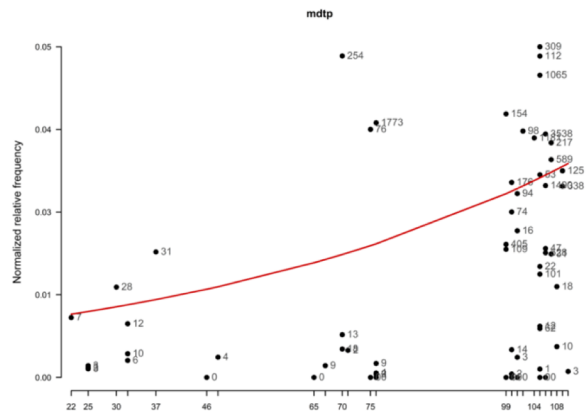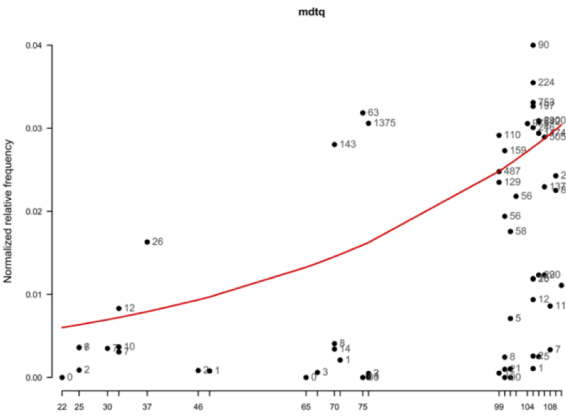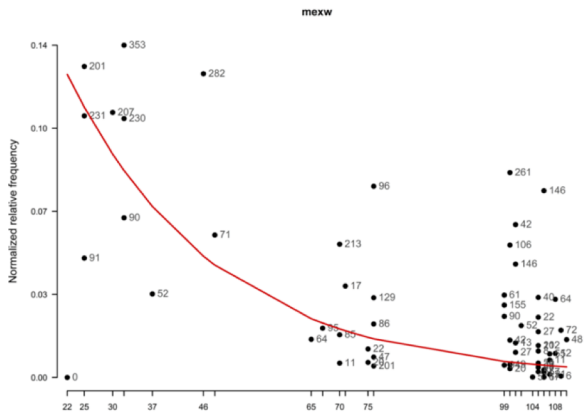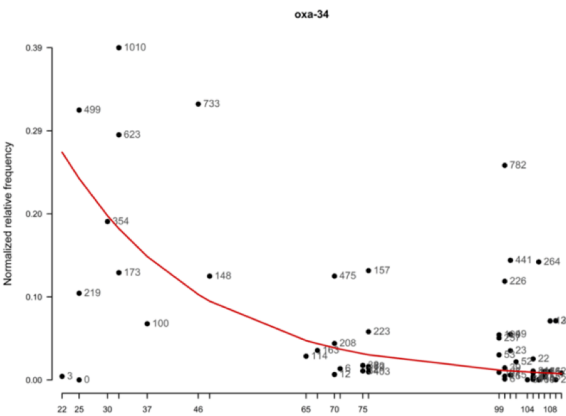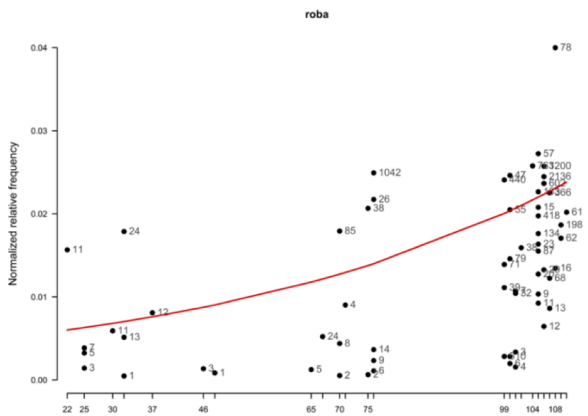

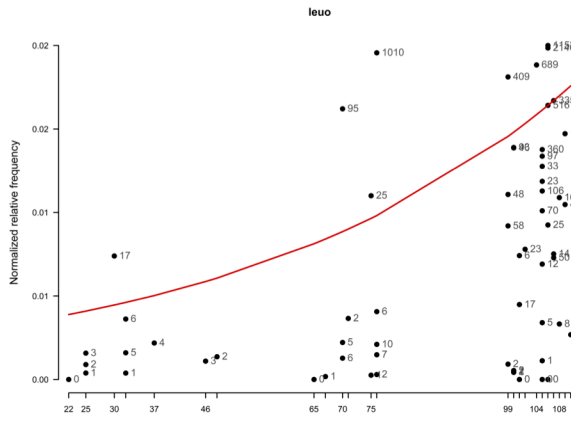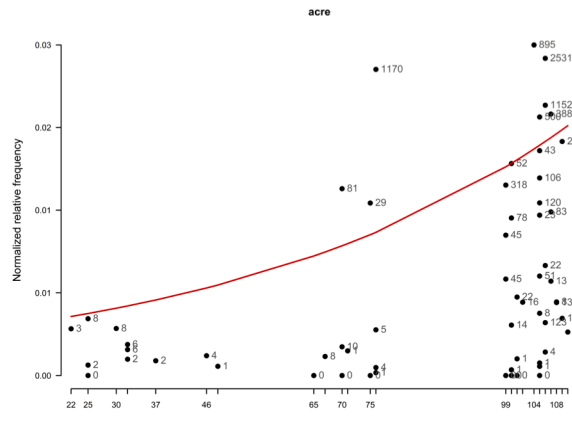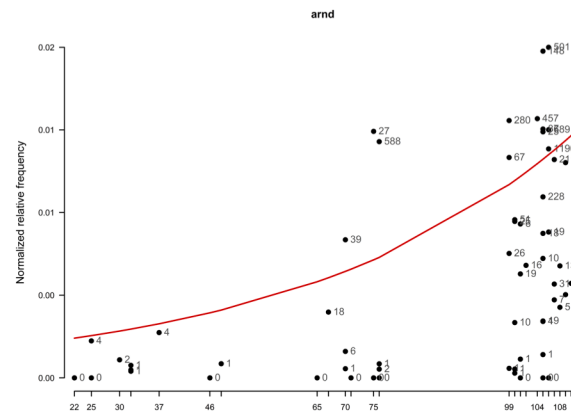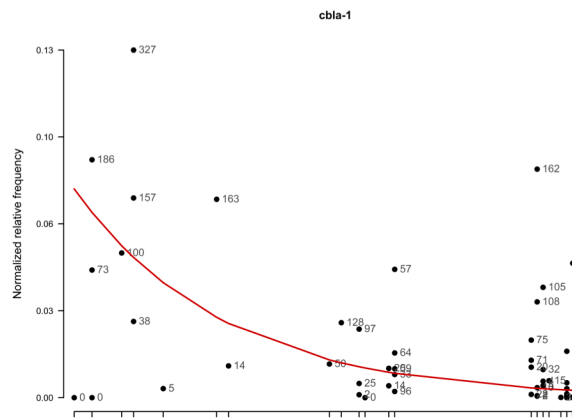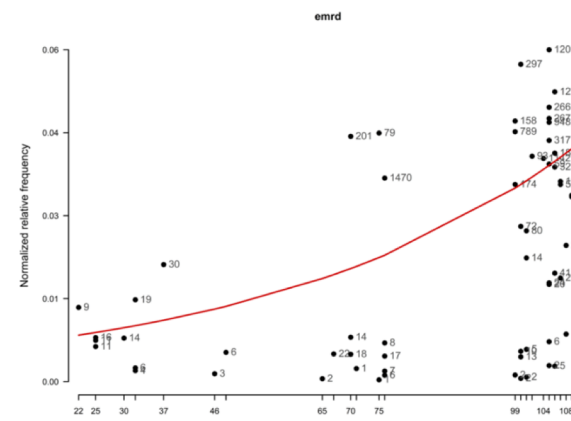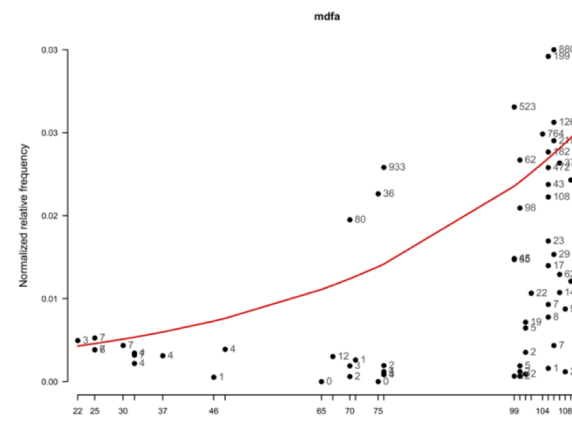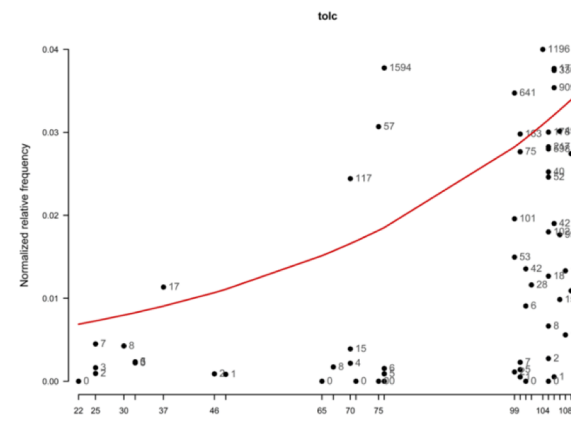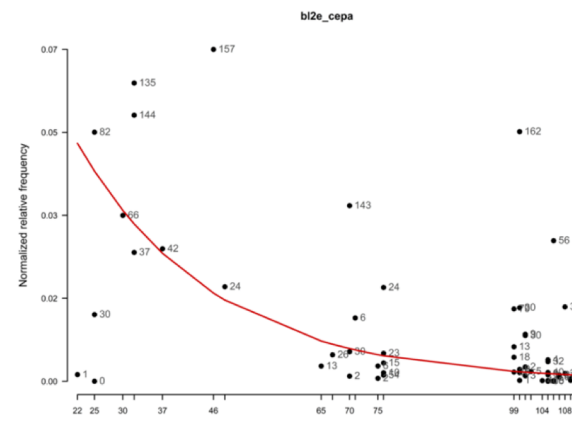

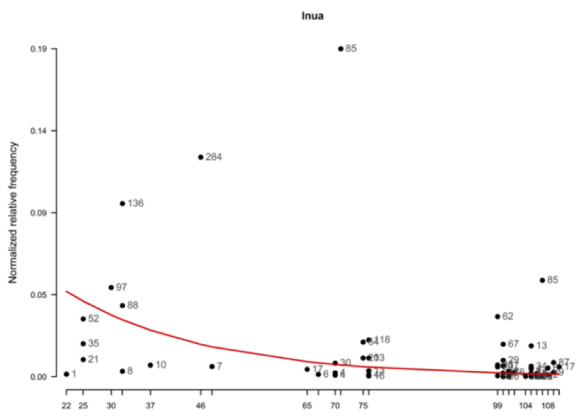

Supplement: FIG S4 [file msphere.00691-21-sf004.pdf]
