# Supplementary material for: The Human Gut Resistome up to Extreme Longevity
Source: mSphere. 2021 Sep 8;6(5):e00691-21. doi: 10.1128/mSphere.00691-21 (PMC8550338; doi:10.1128/mSphere.00691-21)
Supplement: TABLE S4 [file msphere.00691-21-st004.docx]

| **ARD** | **Comparison** | **Coefficient** | **Direction of coefficient (confirmed/**  **not confirmed in DESeq2)** | **P value** | **Drug** |
| --- | --- | --- | --- | --- | --- |
| acrE | Y_vs_S | 2.26 | S ↑(CONFIRMED) | 0.01 | multidrug |
| arnD | Y_vs_S | 2.88 | S ↑(CONFIRMED) | 5.42e-05 | glycopeptide |
| Bl2e_cepa | Y_vs_S  C_vs_S | -3.79  -1.72 | Y ↑(CONFIRMED)  C ↑(NOT CONFIRMED) | 5.20e-06  0.005 | beta_lactam |
| cblA-1 | Y_vs_S | -3.49 | Y↑(CONFIRMED) | 3.06e-05 | beta_lactam |
| emrD | Y_vs_S | 1.72 | S ↑(CONFIRMED) | 0.0003 | multidrug |
| ermB | E_vs_S | -2.28 | E ↑(CONFIRMED) | 0.0005 | macrolide-lincosamide-streptogramin |
| leuO | Y_vs_S | 2.17 | S ↑(CONFIRMED) | 0.0007 | sulfonamide |
| lnuA | Y_vs_S | -3.41 | Y ↑(CONFIRMED) | 0.003 | macrolide-lincosamide-streptogramin |
| mdfA | Y_vs_S | 2.14 | S ↑(CONFIRMED) | 0.0002 | multidrug |
| mdtG | Y_vs_S | 1.99 | S ↑(CONFIRMED) | 0.02 | multidrug |
| mdtH | Y_vs_S | 2.32 | S ↑(CONFIRMED) | 0.0001 | multidrug |
| mdtL | Y_vs_S | 2.53 | S ↑(CONFIRMED) | 0.0002 | multidrug |
| mdtP | Y_vs_S | 1.79 | S ↑(CONFIRMED) | 0.02 | multidrug |
| mdtQ | Y_vs_S | 1.99 | S ↑(CONFIRMED) | 0.0002 | multidrug |
| mexW | Y_vs_S | -3.3 | Y ↑(CONFIRMED) | 1.65e-08 | multidrug |
| OXA-34 | Y_vs_S  C_vs_S | -3.77  -1.77 | Y ↑(CONFIRMED)  C ↑(NOT CONFIRMED) | 5.81e-07  0.001 | beta_lactam |
| robA | Y_vs_S | 1.54 | S↑(CONFIRMED) | 0.0009 | multidrug |
| tolC | Y_vs_S | 2.43 | S ↑(CONFIRMED) | 0.0003 | multidrug |
