# Supplementary material for: The Human Gut Resistome up to Extreme Longevity
Source: mSphere. 2021 Sep 8;6(5):e00691-21. doi: 10.1128/mSphere.00691-21 (PMC8550338; doi:10.1128/mSphere.00691-21)
Supplement: TABLE S5 [file msphere.00691-21-st005.docx]

| **ARD** | **Coefficient** | **P value** |
| --- | --- | --- |
| bcr | 0.018 | 4.352e-07 |
| mdfA | 0.022 | 5.460e-07 |
| mdtH | 0.022 | 1.1760e-06 |
| mexW | -0.038 | 1.545e-06 |
| emrD | 0.018 | 2.1349e-06 |
| mdtA | 0.016 | 4.1229e-06 |
| mdtB | 0.013 | 5.0165e-06 |
| mdtL | 0.022 | 5.1141e-06 |
| arnD | 0.020 | 2.313e-05 |
| mdtQ | 0.018 | 2.4196e-05 |
| emrB | 0.016 | 3.765e-05 |
| robA | 0.015 | 5.668e-05 |
| mdtG | 0.023 | 8.488e-05 |
| OXA-34 | -0.040 | 0.0002 |
| tolC | 0.018 | 0.0002 |
| cblA-1 | -0.039 | 0.0004 |
| mdtC | 0.011 | 0.0005 |
| Bl2e-cepa | -0.041 | 0.0005 |
| leuO | 0.0171 | 0.0007 |
| mdtN | 0.0147 | 0.0008 |
| acrD | 0.011 | 0.0009 |
| lnuA | -0.04 | 0.007 |
| ermF | -0.035 | 0.013 |
| acrE | 0.016 | 0.024 |
| mdtP | 0.013 | 0.042 |
