# Supplementary material for: The Human Gut Resistome up to Extreme Longevity
Source: mSphere. 2021 Sep 8;6(5):e00691-21. doi: 10.1128/mSphere.00691-21 (PMC8550338; doi:10.1128/mSphere.00691-21)
Supplement: TABLE S1 [file msphere.00691-21-st001.docx]

|  |  |  |  | **Therapy** | | | | |
| --- | --- | --- | --- | --- | --- | --- | --- | --- |
| **ID** | **Age group** | **Age (years)** | **Gender** | **Antibiotic** | **Cardiovascular** | **Antihypertensive** | **Hypoglycemic** | **Hypolipidemic** |
| Y2 | Y | 48 | M |  |  |  |  |  |
| Y3 | Y | 25 | F |  |  |  |  |  |
| Y4 | Y | 32 | F |  |  |  |  |  |
| Y5 | Y | 30 | F |  |  |  |  |  |
| Y6 | Y | 32 | M |  |  |  |  |  |
| Y7 | Y | 46 | F |  |  |  |  |  |
| Y12 | Y | 22 | M |  |  |  |  |  |
| Y13 | Y | 37 | F |  |  |  |  |  |
| Y14 | Y | 25 | M |  |  |  |  |  |
| Y15 | Y | 32 | M |  |  |  |  |  |
| Y16 | Y | 25 | F |  |  |  |  |  |
| K300 | E | 75 | F |  |  | X |  | X |
| K301 | E | 70 | F |  |  |  | X | X |
| K303 | E | 71 | F |  |  | X |  |  |
| K304 | E | 76 | M |  |  |  |  |  |
| K306 | E | 76 | F |  |  | X | X | X |
| K124 | E | 63 | F |  |  |  |  |  |
| K119 | E | 70 | F |  |  | X |  |  |
| K105 | E | 75 | M |  | X | X |  | X |
| K100 | E | 75 | M |  |  | X |  | X |
| K125 | E | 71 | F |  | X | X |  | X |
| K113 | E | 65 | M | X | X | X |  |  |
| K106 | E | 70 | M |  |  |  |  |  |
| K108 | E | 76 | M |  |  |  |  | X |
| C010 | C | 101 | F | X |  | X |  |  |
| C012 | C | 100 | F |  | X | X |  |  |
| C042 | C | 102 | F |  | X | X |  |  |
| C052 | C | 104 | M |  | X | X |  |  |
| C021 | C | 101 | F |  |  |  |  |  |
| C048 | C | 99 | F |  | X |  |  |  |
| C002 | C | 100 | F |  | X |  |  |  |
| C003 | C | 99 | F |  | X | X |  |  |
| C004 | C | 98 | F |  | X |  |  |  |
| C007 | C | 99 | F |  |  |  |  |  |
| C017 | C | 101 | F |  | X | X |  |  |
| C018 | C | 100 | F |  | X | X |  |  |
| C024 | C | 100 | F |  | X |  |  |  |
| C054 | C | 100 | F |  |  | X |  |  |
| C011 | C | 99 | F |  | X | X |  | X |
| S010 | S | 107 | F |  |  |  |  |  |
| S020 | S | 105 | F |  |  |  |  |  |
| S030 | S | 105 | M |  |  |  |  |  |
| S050 | S | 109 | F |  |  | X |  |  |
| S080 | S | 109 | F |  |  | X |  |  |
| S100 | S | 107 | F |  | X | X |  |  |
| S110 | S | 105 | M |  |  |  |  |  |
| S120 | S | 107 | F |  | X |  |  |  |
| S130 | S | 106 | F |  | X | X |  |  |
| S140 | S | 106 | M |  |  | X |  |  |
| S150 | S | 105 | M |  |  | X |  |  |
| S180 | S | 106 | M |  |  |  |  |  |
| S190 | S | 105 | F |  | X | X |  |  |
| S200 | S | 105 | F |  |  | X |  |  |
| S210 | S | 107 | F |  | X | X |  |  |
| S220 | S | 108 | F |  |  | X |  |  |
| S240 | S | 105 | M |  | X | X | X |  |
| S260 | S | 105 | F |  |  |  |  |  |
| S280 | S | 105 | F |  |  |  |  |  |
| S290 | S | 104 | F |  |  | X |  |  |
| S300 | S | 106 | F |  |  |  |  |  |
| S320 | S | 105 | F |  |  | X |  |  |
| S330 | S | 109 | F |  | X | X |  |  |
